# The Effects of the LiiNK Intervention on Physical Activity and Obesity Rates among Children

**DOI:** 10.3390/ijerph21101304

**Published:** 2024-09-30

**Authors:** David Farbo, Yan Zhang, Robyn Braun-Trocchio, Deborah J. Rhea

**Affiliations:** Kinesiology Department, Texas Christian University, Fort Worth, TX 76129, USA; david.farbo@cookchildrens.org (D.F.); yan.zhang@tcu.edu (Y.Z.); r.trocchio@tcu.edu (R.B.-T.)

**Keywords:** physical activity, obesity, body composition, accelerometers, MVPA, recess intervention, elementary children

## Abstract

Background: Obesity and inactivity among children are at an all-time high and have been steadily increasing in prevalence over the last thirty years. The school environment provides the ideal setting for reaching a large number of children across diverse populations in order to reverse these trends. However, there are many inconsistent results yielded by school-based physical activity interventions due to implementation length, time for activities, and the use of structured physical activities. The LiiNK Project^®^ is a whole-child intervention addressing these gaps by providing children 45–60 min of recess (unstructured, outdoor play) in their schools daily, while the control children are allowed to engage in recess for 30 min daily. The purpose of this study was to compare the physical activity intensity and obesity rates of third- and fourth-grade children participating in the LiiNK intervention, which provides 60 min of recess for third graders and 45 min for fourth graders, to those in a control group allowed 30 min of daily recess. Methods: The children were 8–10 years old (M = 9.2; 52% females and 48% males). The intervention children comprised 90 third graders and 100 fourth graders, and the control children comprised 101 third graders and 92 fourth graders. Physical activity levels were monitored using accelerometers to assess sedentary, light, and moderate-to-vigorous physical activity (MVPA). Obesity rates were evaluated using bio-electrical impedance analysis (BIA), in which body fat percentage is calculated based on normative values using age and sex in the equation. Results: The third-grade intervention children engaged in 13 more MVPA minutes and took 900 more steps daily than their control counterparts, and also presented a greater proportion of overweight children transitioning to a healthy weight status from the fall to the spring semester. Conversely, the fourth-grade control children increased their activity by 500 steps and 15 more MVPA minutes daily. Despite this, the intervention children overall demonstrated a reduction in body fat percentage, while the control children demonstrated an increase in body fat percentage. Conclusions: Ultimately, 60 min of unstructured, outdoor play in schools provides children the best opportunity to engage in MVPA, which may positively impact body fat percentages, offering a potential strategy for combatting childhood obesity in school settings.

## 1. Introduction

Childhood obesity has reached epidemic levels worldwide as there are currently 340 million children who are categorized as overweight or obese [1,2]. In the United States, it has been estimated there are 14.4 million obese children, with millions more who are overweight [3]. Differences relating to sex and race also present a problem, as male (20.5%), Black (24.2%), and Hispanic (25.6%) children have higher obesity rates than female (18%) and non-Hispanic White children (16.1%) [4]. The health complications that burden obese individuals can include type II diabetes, cardiovascular disease, sleep apnea, and hypertension [3]. Obese individuals can also develop mental health ailments including low self-esteem, reduced quality of life, poor social relationships, and depression [5]. Even more concerning, obese children are likely to become obese adults, leading to life-long health complications [6].

Obesity occurs when more calories are consumed than burned on a consistent basis, leading to excessive body fat accumulation that presents an increased health risk [7,8]. Among children, this caloric imbalance begins with an increase in sedentary behaviors and poor eating habits [9,10]. Most children will spend up to eight hours a day sedentary, and their diets consist of high quantities of snacks, sugary beverages, and fast food; additionally, they frequently engage in over eating [9,10]. Physical activity (PA) has been shown to be an effective way to burn excess calories and prevent body fat accumulation [11]. The CDC recommends that children engage in at least 60 min of moderate to vigorous physical activity (MVPA) daily to prevent weight gain and even decrease body fat [12]. However, only 24% of children meet this recommendation, with females achieving 10–20% less MVPA daily than males [12,13,14]. In addition, children who are inactive will continue to be inactive as adolescents and adults [15]. This life-long risk of inactivity develops as a result of children never learning how to adopt an active and healthy lifestyle [16].

If given the opportunity, children will naturally learn how to be physically active through unstructured or structured play. Unstructured play is child-directed and child-controlled, with no specific end goal needed [17,18,19]. Structured play can include sports or games that are planned and adult-led with a specific end goal in mind [18]. Unstructured, outdoor play provides more opportunities for physical activity than structured play. In fact, children are physically active about 40% of the time, especially when outdoors and engaged in unstructured play [20]; in contrast, they are physically active less than 25% of the time during structured play [21]. Most importantly, children enjoy unstructured play and will participate regardless of any adult guidance, developing their motivation to regularly engage in this behavior. Unfortunately, many environmental factors, i.e., safety and distance to parks, can limit the amount of time children have to engage in sufficient unstructured, outdoor play [19,22,23].

Schools are the most logical places in which all children have opportunities to be physically active, but this quality has become more limited over the past few years due to an over-emphasis on academic content, i.e., math and reading, and less balance with recess (unstructured, outdoor play) and physical education (structured play) throughout the school day [19,23,24]. When schools do schedule recess, it can account for up to 40% of a child’s daily MVPA levels [25]. Providing the minimum amount of recess (100 min per week) is also associated with a slight decrease in body mass index (BMI) scores compared to those children who do not have recess [26]. Without recess, females have greater odds of becoming overweight or obese than males [27]. Evidence suggests recess is vital to increase PA and decrease obesity rates among children.

While several studies have implemented various recess intervention strategies and focused on structured play activities to improve MVPA and decrease obesity rates among third- through sixth-grade children, very few have shown significant positive outcomes [28,29]. The limitations of these prior studies could explain why these interventions were not effective. First, 30 min of recess may be insufficient to produce significant BMI score decreases [30,31,32]. Some studies have shown up to 60 min of recess time is needed in preschool through late elementary grades to realize any decline in BMI scores [26,33]. However, it seems adding shorter, more frequent play breaks may be the key to more consistent MVPA increases and obesity percentage declines [34]. Finally, when children are more active, they may burn more fat and gain more muscle mass, which means a more valid and reliable body fat percentage assessment tool is needed in the field instead of BMI and skinfold assessments [35,36].

The LiiNK (Let’s inspire innovation ‘N Kids) Project intervention provides answers to the above limitations for grades K-5 children. Typically, four 15 min recesses, defined as unstructured, outdoor play, are provided throughout the day, with no removal for punishment or tutoring [19,37]. Unstructured play is defined as being self-controlled and self-directed. The unique aspect of this definition is that physical activity is not the central focus of play. There is no expectation of active play. Oddly enough, in this play environment, more moderate-to-vigorous active play takes place than when structured or semi-structured recess is implemented or when less recess is offered daily [38,39]. This intervention has shown the following positive results: (1) first- and second-grade children took 900 more steps daily and engaged in 25 more minutes of MVPA daily than the control school children, who were provided 30 min of recess daily [38]; (2) bio-electrical impedance analysis (BIA) is more reliable and valid for assessing children’s body fat percentages in the field than body mass index (BMI) [39]; and (3) obesity rates among second-grade children and females declined over a year when allowed 60 min of recess daily [40]. One interesting LiiNK Project finding is that obesity percentages and shift differences from obesity to overweight or healthy weight were not found for children who engaged in at least 30 min or more of recess daily [40]. The past few studies’ results focused on first- and second-grade children, so focusing on the PA patterns and body composition results of third- and fourth-grade children is the next step.

Therefore, the purpose of this study was to examine PA and body composition among third- and fourth-grade children who engaged in 30–60 min of recess daily. The first research question examined differences in steps and MVPA between groups by sex and grade among children who engaged in 30, 45, or 60 min of unstructured, outdoor play. PA intensity was measured by minutes spent engaging in sedentary, light, and moderate-to vigorous physical activity throughout the school day. The intervention children participated in 45 or 60 min of recess daily, while the control children had no more than 30 min daily. It was hypothesized that the intervention third- and fourth-grade children would have more MVPA minutes and steps than the control children. The second research question examined body fat category shift differences between the intervention and control children by sex and grade. The body fat categories were classified as underweight, healthy, overweight, or obese based on BF%, age, and sex. Category shift was identified by examining the body fat category from the pre- (Sept) to post (March)-assessments. It was hypothesized that the intervention third- and fourth-grade children would shift from the overweight/obese category to the healthy category at a greater extent than the control children.

## 2. Materials and Methods

### 2.1. Study Design

A control group posttest-only design, also known as a static group comparison design, was used to answer the first research question and determine differences in PA between the intervention and control groups. Pretest measurements could not be taken since the children in both groups had already been allowed more minutes for recess when data collection occurred. Accelerometers were used to determine differences between intervention and control groups in terms of the number of steps and MVPA minutes throughout the day. A pretest–posttest non-equivalent groups design was used to answer the second research question examining differences in body composition between pre- and post-measurements. Body composition was measured using BIA, in which BF% was used to categorize students as either underweight, healthy, overweight, or obese.

Children from three elementary schools (N = 383) across two districts located in either North or South Central Texas participated in this study (Table 1). District 1 (N = 190) included 3rd- and 4th-grade children from two North Texas elementary schools engaged in the LiiNK intervention (I), while District 2 (N = 193) included 3rd- and 4th-grade children from two South Central Texas elementary schools labelled the control (C) schools. The intervention school third graders were allowed three 20 min recesses, while the intervention school fourth graders were allowed three 15 min recesses. The recess minute differences were due to administrators and teachers wanting to spend more time in the 4th-grade classrooms to make up for the lack of learning during the COVID-19 pandemic. Control school children engaged in two 15 min recesses in both grades. The reason for using one intervention school and two control schools was to collect a more equal number of children according to group and grade.

Intervention children participated in recess either on a traditional playground, a bus loop, or a blacktop. The traditional playground included playground equipment (slides, swings), a grassy area, and a hill. The bus loop contained a grassy area, blacktop, stones and logs, and a large musical instrument section. The blacktop was in the back parking lot of the school and did not include any equipment. The control school play environment included traditional playground equipment, grassy areas, a climbing net, and a gaga ball pit. Their playgrounds were recently redesigned to standardize the play environments across the campuses.

### 2.2. Participants 

The composition of the District 1 intervention group was approximately 40% White, 40% Hispanic, 15% Black, and 5% other, while the District 2 control group’s composition was approximately 70% Hispanic, 20% White, 5% Black, and 5% other. The ‘other’ category for both districts consisted of Asian, Pacific Islander, American Indian, Alaskan Native, and two more races. Students were excluded if they wore a pacemaker or other device that did not allow their body fat data to be collected. They were also excluded if they had a physical disability that limited their recess movement/physical activity.

A priori power analysis was conducted using G*Power 3.1.9.2 prior to data collection to determine the appropriate sample size to employ an ANOVA statistical method for the first research question with a medium effect size (Cohen’s ƒ^2^ = 0.0625) and an alpha of 0.05. The power analysis determined that a sample size of N = 197 was needed to achieve a power of 0.8 [41]. As a result, 200 children (I = 100, C = 100) were recruited to have their PA data collected. However, 44 children (I = 16, C = 28) did not meet the minimum wear time of at least five school days with 360 min of data collection. In addition, two of the intervention school children misplaced their accelerometers; therefore, their data could not be recovered. Therefore, 154 children (I = 82, C = 72) were included to determine PA differences between intervention and control groups, with a further breakdown shown in Table 1.

A larger sample was collected for body fat (BIA) than the PA intensity data as a result of the accelerometers available and parent consent. Therefore, 454 children (I = 219, C = 235) were originally recruited to participate in this study. Children needed to have both pre- and post-measurements to be included in the final analysis, leading to the elimination of 70 children with unmatched data (I = 28, C = 42). Finally, one intervention child had an error in their profile data as they decreased in height between semesters. The final sample size for body composition data was N = 383 (I = 190, C = 193). A further breakdown of the sample divided by school, grade, and sex can be seen in Table 1.

### 2.3. Measures

#### 2.3.1. Physical Activity

The first measure, PA, was assessed using an Actigraph wGT3X-BT accelerometer to determine steps and minutes spent in PA of various intensities. This device has been shown to be a valid and reliable tool for assessing children’s PA engagement [42]. The accelerometers calculate PA by using tri-axel sensors to detect changes in vertical, horizontal, and perpendicular orientations during different time sampling intervals known as epochs. A 15 s epoch value was set for the current study, as shorter epoch lengths (1 s–15 s) are more accurate for assessing MVPA engaged in by children than longer epochs (30 s–60 s) [43]. During each of these epochs, the device reports raw data in counts per minute (CPM), which are used to validate wear time and the number of steps and time spent engaging in sedentary, light, moderate, or MVPA. In order to have better precision with the epoch length of 15 s, 120 Hz was the data frequency used for this accelerometer type.

Children needed at least five days with 360 min of wear time during school to be included in the final analysis. The device has a wear time validation feature that can determine how long a participant wears it. Consecutive epochs of no movement in which the number of CPM does not change is an indication the participant is not wearing the device. The Actigraph [44] wear time validation parameters were used, and 30 min or more of consecutive epochs with no movement was considered non-wear time.

A scoring system was used to convert CPM into minutes spent in each of the different PA categories. Previous LiiNK intervention studies examining PA used the Puyau [45] children-scoring system, which classifies sedentary as 0–799 CPM, light as 800–3199, and moderate to vigorous as 3200 and above. To accurately compare the results of this study with prior LiiNK research [38], the Puyau [45] children-scoring system was used.

#### 2.3.2. Body Fat Assessment

Bio-electrical impedance analysis scales (BIA) were used to assess each child’s body fat percentage in the school setting, as performed in previous studies [38,46]. The scale used for this study was the TANITA BF-2000, which has high specificity and sensitivity in regard to identifying overweight and obese children [46]. The scale uses small, unnoticeable electrical signals sent throughout the body via foot-to-foot metal plates located at the bottom of the device. Due to body fat having a low water content, it will cause resistance in the current, which the scale uses to determine BF%, fat mass, and fat free mass. Children are then categorized based on their BF% as either underweight (1), healthy (2), overweight (3), or obese (4) based on McCarthy et al.’s [47] normative values. These child normative values take age and sex into consideration, so the range of BF% in these categories can vary between children. This scale does not contain any screens, and all data were uploaded to a computer from the BF-2000 Ironkids software via Bluetooth. Children were blind to their results, which is an important factor to consider as knowing one’s weight status could have negative psychological effects.

To examine whether the children had improved in the body fat category from the pre- to post-assessment periods, a new variable was created to determine a shift by subtracting the pre-assessment body fat category value from the post-assessment body fat category value. A value of −1 indicated a participant exhibited a decrease in a body fat category, while 1 indicated an increase, and 0 indicated no difference. For example, if a child shifted from being obese to overweight, they would be coded as a “−1” since they exhibited a decrease in a given body fat category. If a child shifted from being healthy to overweight, they were coded as a “1” since they exhibited an increase in a body fat category. While these children demonstrated an increase or decrease in BF%, they were not at risk of developing obesity. No child shifted to more than one body fat category between pre- and post-measurement periods.

### 2.4. Procedures

#### 2.4.1. Physical Activity

For PA data collection, a rotation was implemented so the 3rd-grade children from the three schools could wear the devices simultaneously, followed by the 4th-grade children. The 3rd-grade children wore them for the first two weeks in September and March, followed by the 4th-grade children wearing them for the next two weeks. Before starting collection, the devices were programmed and assigned an ID number to track the student’s data throughout the collection period.

On the first day of collection for each grade, researchers were present to distribute the assigned numbered device to each child and explain to the teachers and the children the procedures for each day of the two-week period. Children were instructed to wear their assigned numbered device on their non-dominant wrist from the time they entered school in the morning (7:30 for the intervention, 8:30 for the control) until they left in the afternoon (2:30 for the intervention, 3:30 for the control) each day for two weeks. Although the intervention and control school day times were not equal, a wear time correction was made by setting the intervention school daily wear time to begin at 7:50 am and conclude at 2:20 pm, while the control schools were set to begin at 8:35 and end at 3:15 pm. Teachers were instructed to make sure the children received the correct accelerometer from the storage case each morning, as identified on the class roster, and the accelerometer was placed back into the correct slot of the storage case at the end of the day. The accelerometers were stored in the teachers’ locked file cabinets at the end of each day.

The range of wear-time days among the children was from five to nine days. Regardless of the total wear day number, an average number of CPM for each day was used in the final analysis. The children repeated these procedures daily for two weeks and did not wear the devices at home or on the weekend. Before collecting the data from the fourth graders, the devices were charged and reprogrammed. Data collection concluded and all accelerometer devices were collected on Friday of week 4 from the three schools in the fall and the spring.

Teachers were asked to track children who were absent on data collection days or provide feedback on any hindrances to their normal school schedule. These changes in schedule included half days, bad-weather days, and, in one instance, a school did not have any power for half of the day, so the children did not wear the accelerometers. Any days with these problems were excluded from the final analysis.

#### 2.4.2. Body Fat Assessment

BIA assessment days were set in collaboration with the principal and physical educator of each school, and the assessment time was scheduled to occur during the physical education class. A height, weight, and body fat (BIA) station was set up in a private area close to the gym so each child felt comfortable and their peers could not see them during the assessment. All children were informed of the task to be completed after parent consent was obtained. Once the children assented, a group of 10–15 children was instructed to line up in the hallway, remove their socks and shoes, and wait for their names to be called.

Once called, each child stood next to a measuring tape located on the wall so that their height could be recorded in inches into the software program; then, they were asked to step on the BIA scale to have their weight and body composition measurements recorded. Each child stood with their feet placed on the metal plates of the scale for about 15–20 s to complete the process. Once the procedure was completed, each child stepped off of the scale and went back to the gym while the scale was disinfected, and the next child was called to repeat the same procedures. Each class assessed took approximately 30–40 min to complete the process depending on class size and efficiency of the data collection.

All data, including weight, BF%, and body fat category, were automatically saved to the BIA software on the laptop computer after each measurement. At the end of each day, all data were downloaded and saved to a master data Excel file. The intervention school required three days of data collection, while the control schools each required one day. These procedures were utilized for the pre- and post-data collections.

### 2.5. Data Analysis

Data were analyzed using IBM SPSS version 28. Descriptive statistics were determined to describe the means and standard deviations for weight, BF%, steps, and minutes engaging in sedentary, light physical activity, and MVPA for groups according to sex and grade. To answer the first research question, a multivariate analysis of variance (MANOVA) was used to examine group differences (intervention and control) according to sex and grade. If there were any interactions, a two-way ANOVA was used to determine any significant differences. Assumptions of MANOVA, including the absence of multicollinearity and multivariate normality, were checked and met. Multicollinearity was assessed using Variance Inflation Factors (VIFs) and tolerance values, while multivariate normality was evaluated using Shapiro–Wilk tests and Q-Q plots. Although the assumption of the homogeneity of covariance matrices was violated, Pillai’s Trace statistic was used, which is robust under such violations.

To answer the second research question, non-parametric chi-squared tests were used to assess any associations between body fat category shift and group, sex, or grade respectively. An alpha value was set at *p* < 0.05. If there was a significant chi-squared result, it indicated there was an association between group, sex, or grade and body fat category shift. Adjusted standardized residuals (ARs) were used to further determine significant differences between the observed and expected values. An AR greater than 1.96 indicated a significant difference between the observed and expected values for this particular category.

## 3. Results

### 3.1. Research Question 1: Hypothesis 1

A 2 × 2 × 2 MANOVA was used to determine steps and average minute differences with respect to PA intensities between groups by sex and grade for Hypothesis 1. All MANOVA assumptions were met, except the assumption of homogeneity of covariance matrices. As a result, Pillai’s Trace statistic was used to interpret the results. The MANOVA revealed a significant three-way interaction effect for group by sex and grade (Pillai’s Trace = 0.07, F(4, 143) = 2.809, *p* = 0.03, $\eta_{p}^{2}\;$= 0.07), a two-way interaction for group and grade (Pillai’s Trace = 0.26, F(4, 143) = 12.31, *p* < 0.001, $\eta_{p}^{2}\;$= 0.26), and main effects for group (Pillai’s Trace = 0.28, F(4, 143) = 14.11, *p* < 0.001, $\eta_{p}^{2}$ = 0.28), sex (Pillai’s Trace = 0.28, F(4, 143) = 14.06, *p* < 0.001, $\eta_{p}^{2}\;$= 0.28), and grade (Pillai’s Trace = 0.71, F(4, 143) = 87.37, *p* < 0.001, $\eta_{p}^{2}\;$= 0.71).

A between-subjects test for the three-way interaction of group by sex and grade revealed significant differences for steps (F(1, 146) = 8.06, *p* = 0.005, $\eta_{p}^{2\;}$= 0.05) and MVPA (F(1, 146) = 6.54, *p* = 0.01, $\eta_{p}^{2}\;$= 0.04). Male third graders in the intervention group took significantly more steps (1483) than the control school males (*p* < 0.001, $\eta_{p\;}^{2}$= 0.44). Additionally, male third graders in the intervention group had significantly more MVPA minutes (25) than the control third-grade males (*p* ≤ 0.001, $\eta_{p}^{2}\;$= 0.27). Among males, these results support the first hypothesis that children given 60 min of recess would have more steps and MVPA minutes than children given 30 min of recess. There were no other significant steps or MVPA differences in the three-way interaction among group, sex, and grade.

The significant group–grade interaction differences were in relation to steps (F(1, 146) = 29.94, *p* ≤ 0.001, $\eta_{p}^{2}$ = 0.17), sedentary (F(1, 146) = 14.90, *p* ≤ 0.001, $\eta_{p}^{2}\;$= 0.09), light PA (F(1, 146) = 5.02, *p* ≤ 0.03, $\eta_{p}^{2}\;$= 0.03), and MVPA (F(1, 146) = 19.52, *p* ≤ 0.001, $\eta_{p}^{2}\;$= 0.11). The third-grade intervention children had significantly more steps (843) and MVPA minutes (13) than the control school children (*p* ≤ 0.001, $\eta_{p}^{2}\;$= 0.17; *p* = 0.02, $\eta_{p}^{2}\;$= 0.08). The control third-grade children had significantly more sedentary minutes (14) than the intervention children (*p* = 0.04, $\eta_{p}^{2}\;$= 0.05). These results support the first hypothesis that children provided 60 min of recess will take significantly more steps and engage in more MVPA minutes than those provided 30 min. In fourth grade, the control children took significantly more steps (524) and had more light-PA minutes (11) and MVPA minutes (16) than the intervention children (*p* = 0.01, $\eta_{p}^{2}\;$=.08; *p* ≤ 0.001, $\eta_{p}^{2}\;$= 0.16; *p* ≤ 0.001, $\eta_{p}^{2}\;$= 0.15). The fourth-grade intervention children had significantly more sedentary minutes (19) than the control children (*p* = 0.001, $\eta_{p}^{2}\;$= 0.13). These results rejected the first hypothesis that children provided 45 min of recess would have more steps and MPVA minutes than children provided 30 min. Table 2 provides the means and standard deviations for steps and minutes in sedentary, light activity, and MVPA, as well as the significant interaction effects.

### 3.2. Research Question 2

#### Body Fat Descriptive Changes and Hypothesis 2

Three non-parametric chi-squared tests were used to answer the second hypothesis and determine if the body fat category shifts observed were different between groups by sex and grade. The chi-square assumptions were met for each test. The grade-level results in the first two bullets show descriptive differences. The last three bullets refer to the second hypothesis and show no differences between groups, therefore showing the intervention did not impact body fat shifts from baseline to the follow-up time. Table 3 shows all of the bulleted results highlighted below.

- Third Grade: The intervention group males exhibited a 17% BF decrease; in contrast, the control males exhibited an 82% BF decrease. The intervention group females, however, exhibited a 56% BF increase; in contrast, the control females exhibited a 38% BF increase.
- Fourth Grade: Males and females in the intervention group exhibited 43% and 19% decreases in BF%, respectively, whereas the control group males and females showed 49% and 23% increases in BF%.

Obesity and Overweight Category Changes

- In the intervention group, a slight increase in the obesity category (4%) was noted among males, with a 12% reduction in the overweight category. The control group males exhibited a 2% decrease in obesity levels, and there was no change among females.
- In the fourth-grade cohort, there was an increase in the overweight category for intervention males (2%), while a decrease was noted among intervention females (6%) and control males (17%) and females (11%).
- The chi-squared tests showed no significant differences between the intervention and control groups regarding shifts in body fat categories across sex and grades, suggesting consistent body fat category shifts irrespective of the intervention (χ^2^(2) = 0.71, *p* = 0.70).

## 4. Discussion

Previous research has shown when engaging in up to 60 min of MVPA daily can prevent excessive body fat accumulation and decrease a child’s chances of becoming overweight or obese [11,12]. Farbo et al. [38] found that first- and second graders take more steps and have more MVPA minutes when allowed 60 min of recess than those allowed 30 min of recess daily. Farbo and Rhea [40] then noted some interesting body composition trends among second–fifth-grade children who engaged in 30–60 min of recess, but accelerometer data were needed to verify whether these differences were a result of increased time for recess. To advance both study results further, the current study sought to assess third- and fourth-grade children, with a focus on two things: (1) Are there differences in steps and MVPA between groups of children who engage in 30, 45, or 60 min of unstructured, outdoor play? (2) Will overweight/obese rates decrease as a result of increased minutes spent in play daily? Sixty minutes of unstructured, outdoor play daily was only provided for the third-grade intervention children. The fourth-grade intervention group had 45 min of recess, and both control school grades had 30 min.

Unsurprisingly, the intervention children in third grade with 60 min of recess were the most active group when compared to all other children. Intervention children in the third grade took ~900 more steps and had ~13 more MVPA minutes than the control children. Other interventions that implement up to 30 min of structured or unstructured recess only show a 0–5 min MVPA group difference in post-measurements, and this amount of time often does not result in significant body composition changes [31,48,49]. While obesity percentages did not change, the increase in MVPA resulted in more intervention third graders shifting from overweight to healthy than the control third graders. This is significant considering overweight children have a high obesity risk if they do not make immediate changes in their lifestyle habits. If they become obese at a young age, they are likely to remain obese as adolescents and adults, leading to lifelong health complications [6]. The PA patterns among the third-grade intervention children in the current study are similar to first- and second-grade children in the LiiNK project who also engaged in 60 min of unstructured, outdoor play [38]. The children in both of these studies achieved higher quantities of steps and MVPA minutes when compared to children who were only allowed 30 or 45 min [38]. Based on these collective results, it is clear that 60 min of recess is needed for children to achieve the most significant improvements in MVPA, which can then lead to positive body composition changes.

As a result of decreasing recess to 45 min, the intervention fourth-grade children were less active than the third-grade intervention children and consequently exhibited fewer body fat percentage shifts to a healthy state. Although this may be a result of fewer provided minutes, it may also be a result of variables that we did not think would impact PA, including recess space design and play preference differences across age groups. The space was the same for the control school children, but the intervention fourth graders played in a closed-off parking lot with very few loose parts to engage with, while the intervention third graders played on a large field and playground where they had lots of engaging equipment. In addition, other limitations such as the time for physical education and school layout may also explain why the fourth-grade control children had slightly more steps and MVPA minutes than the intervention children. Even with less MVPA, the intervention children in the fourth grade actually had decreased BF%, while the control children had increased BF%. The control school children mirrored what other recess interventions have shown when implementing 15–30 min of structured or unstructured recess, whereas the intervention children showed improvements when having access to play for 45 to 60 min [30,31]. Two conclusions can be drawn from these fourth-grade group differences. First, there may be outside factors such as eating and PA patterns outside of school that could have led to this result since the intervention children had lower levels of MVPA than the control children. Second, there may be greater longitudinal body composition changes when children are given additional time for recess, as this was the fifth year the intervention children were allowed 45–60 recess minutes. The fourth grade may be the “tipping point” where BF% begins to decrease at a higher rate from the consistent increase in MVPA [30]. The fourth-grade intervention group may achieve even greater decreases in BF% if the school personnel continue to offer the full 60 min of recess that the third-grade group received and provide more loose parts to engage the children.

Children who were categorized as obese did not change between pre- and post-measurements regardless of the number of recess minutes received. For obese children, increased recess minutes may be insufficient to elicit body composition changes, and they may need other components such as nutrition education or family involvement [28]. We did not account for these factors since single-component interventions that target MVPA are generally more effective in changing body fat than those focused on other obesity-related behaviors [28,50]. However, we did find that the subgroups that achieved higher MVPA levels (i.e., the third-grade intervention and the fourth-grade control) saw more children shift from being overweight to healthy. Previous research has shown an inverse relationship between more minutes of higher-intensity PA and adiposity among children [51]. We also found that the intervention children had greater odds of being healthy or overweight than obese with every minute increase in vigorous activity. All the children, whether they were provided 30, 45, or 60 min, averaged ~66–129 MVPA minutes during school, which is more than other school-based interventions that provided either structured or unstructured activities and reported up to ~30 min of MVPA daily [28,29,52]. This also means that these children were nearly doubling the recommended 60 min of MVPA while in school simply through additional time for recess. The 60 min group averaged 116–129 min of MVPA, which means they were less likely to be obese than the 30 or 45 min group, which only averaged 66–116 min of MVPA. Since 60 min of recess resulted in the highest averages of vigorous PA, this is the minimum amount of time needed to give children their greatest odds of remaining healthy.

It is clear that 60 min of recess is needed to elicit the greatest improvements in MVPA and body composition, but in this study, females and older children needed more recess to produce healthier results. When comparing our results with prior LiiNK intervention children provided 60 min of recess, it can be observed there is a noticeable decrease in MVPA between the second and third grade, with an even larger decrease for fourth graders provided 45 min of recess [38]. In addition, females are consistently less active than males regardless of age or recess minutes provided [38]. PA among children is shown to peak at about the age of eight and then decrease, with males consistently achieving more MVPA minutes than females [13,53]. This is a problem for both of these populations, as BF% will steadily increase until about age 11, so a lack of sufficient MVPA may lead to excess body fat accumulation and obesity [51]. Females and older children especially need additional time for recess to ensure they engage in enough MVPA to at least prevent the development of obesity. While females and older children still may be less active than males and younger children, 60 min of recess gives them more of an opportunity to be active and achieve healthier body fat levels than 30 or 45 min allow.

The type of play environment also had an effect on children’s MVPA during recess regardless of the available minutes for recess. Children in both grades were significantly more active when recess was provided on the playground compared to the bus loop or blacktop. In these environments, the children were more likely to engage in sedentary activities such as socializing, while on the playground, they were observed to engage in higher-intensity activities such as running and jumping. Other studies have shown that each piece of play equipment available on a playground increases MVPA by 50% [54]. There was insufficient play equipment available on the bus loop or blacktop to result in MVPA levels similar to those observed when children were on the playground. This may explain why the fourth-grade control children had more steps and MVPA minutes, since they were on the same playground for each of their recesses. There may be more significant results in favor of the intervention if both groups had the same play environments for each recess in both grade levels. However, we also need to consider the play needs of different populations of children (i.e., with respect to sex, age, and race) as activities that were popular among one group of children were not as popular in other groups (i.e., boys ran in the field area, while girls walked and socialized). To ensure children are achieving their highest MVPA levels during recess, play spaces need to provide appropriate opportunities to cater to the needs of children across all populations. This could include the addition of more playground equipment (i.e., climbing structures, swings), loose parts (i.e., balls, hula hoops), natural elements (i.e., rocks, hills, climbing trees), or green space [55]. Combining 60 min of recess and the proper play environments may lead to even greater MVPA and body composition improvements in children.

### 4.1. Limitations

A number of limitations need to be addressed for both the accelerometer and BIA portions of the study. The first limitation is the differences in time for physical education (PE), as the control children attended PE three times every five days, while the intervention children only had two PE classes every five days. As many children did not meet the minimum wear time, filtering out days with physical education from those without was not possible. The layouts of the schools were also very different, as the control children had to travel through separate buildings for lunch, specials (PE, music, and art), and recess, while the intervention school was self-contained in one building. This additional PE class and the differences in school layout may have contributed to the greater number of steps and light– and moderate PA minutes noted for the fourth-grade control children. The second limitation is the children needed to be fasted and fully hydrated before the BIA measurements were taken so that they could be collected most effectively. Due to the varying needs of children throughout the day, this aspect could not be completely controlled, but the children were measured at the same time of day at both data collection time points to counter this limitation. Additionally, the accuracy of a participant’s height could also have influenced the results concerning the BIA scale. The researchers ensured the same protocols were used for both groups when measuring height. However, if there was an error in how the measurements were read or how they were entered into the BIA program, it could have resulted in inaccurate measures of BF%. Similar to Farbo et al.’s [40] study, some lingering effects of the COVID-19 pandemic between measurements could have lessened any differences in body composition.

### 4.2. Future Directions

Future research should consider ways to maintain PA among females and older children since there was a noticeable difference in MVPA compared to males and younger children. This may include adding even more time for recess or additional play choices that encourage MVPA. Future research should also examine the PA patterns of children outside of school during the week and on the weekend. To obtain a holistic view of their PA behaviors, combining younger children’s MVPA results with the current findings will help support the need for more recess during the school day to help children attain healthy levels of MVPA. Future research should also examine the effects of recess on obesity in a normal school year that is not affected by COVID-19. Based on the discrepancies between the BIA and accelerometer data in regard to the fourth grade, outside factors could have influenced the obesity rates in this sample of children. Future research should more closely examine how other variables such as diet can be used to elicit greater obesity changes in children. Females in this and previous LiiNK studies seem to have attained more positive results from the additional time for recess than males. Future research should consider what strategies need to be utilized for males to exhibit similar obesity prevalence results. Future research could also consider adding subjective measures to examine the reasons why children engage in or disregard physical activity. Finally, a larger sample size is needed to examine if MVPA is predictive of body fat percentages in LiiNK intervention and control children.

## 5. Conclusions

Sufficient MVPA is needed to help children maintain healthy body fat levels and prevent the development of obesity. Based on our findings, 60 min of unstructured, outdoor play provides children with the best opportunity to engage in a sufficient amount of MVPA to result in positive changes in body composition. This is the minimum amount of time schools and researchers should provide to see the most significant health improvements in children. We also found that available play spaces and equipment can have a significant impact on the amount of MVPA children are able to achieve. Providing both additional time for play and appropriate environments may be the key to shifting the current obesity and inactivity trends seen among children today.

## Figures and Tables

**Table 1:** Children arranged by group, grade, and sex.

		Intervention	Control	
Grade	Sex	N	N	Total
Physical Activity				
3rd	M	21	18	40
	F	24	15	39
4th	M	18	14	35
	F	19	25	46
	**Physical Activity** **Total**	82	72	154
Body Composition				
3rd	M	50	52	102
	F	40	49	89
4th	M	45	37	82
	F	55	55	110
	**Body Composition Total**	190	193	383

**Table 2:** Physical activity means and standard deviations.

			Steps		Sedentary		Light PA		MVPA	
Grade	Sex	Group	Mean	SD	Mean	SD	Mean	SD	Mean	SD
3rd	Males	I	8624.74 ***	898.73	122.13	24.21	147.28	11.33	129.44 ***	21.32
		C	7141.22	793.88	149.05	28.57	146.30	14.96	104.35	20.67
	Females	I	7601.18	760.61	133.14	28.26	148.28	12.71	117.25	22.98
		C	7347.53	957.70	131.43	26.81	151.96	10.65	116.33	24.95
	3rd-Grade Total	I	8078.84 ***	967.59	128.00	26.73	147.81	11.96	122.94 *	22.81
		C	7235.00	864.26	141.04 *	28.77	148.87	13.29	109.80	23.15
4th	Males	I	6343.10	772.13	169.66	25.05	129.91	12.38	82.70	17.15
		C	7148.71	1195.24	148.72	28.56	138.28	14.29	105.20	25.72
	Females	I	5817.58	622.28	176.72	22.89	125.63	9.8	82.95	13.72
		C	6289.52	766.28	157.35	23.13	138.26	12.81	94.70	19.19
	4th-Grade Total	I	6073.24	738.95	173.28 **	23.90	127.71	11.19	82.83	15.27
		C	6597.95 *	1016.83	154.25	25.19	138.26 ***	13.17	98.49 ***	22.02
		Combined Group Total	7041.12	118.35	148.32	31.04	140.79	14.91	104.29	25.80

Note. I = Intervention; C = Control; *** = *p* < 0.001, ** = *p* < 0.01, * *p* < 0.05.

**Table 3:** Body fat % changes from baseline to follow-up.

Group	Grade	Sex	BF% Baseline (Mean ± SD)	BF% Follow-Up (Mean ± SD)	Δ BF%	Obesity Shift	Overweight Shift
Intervention	3rd	Male	22.61 ± 6.98	22.44 ± 7.79	−17%	+4%	−12%
		Female	26.99 ± 9.20	27.55 ± 9.04	56%	No change	−7%
	4th	Male	23.99 ± 8.84	23.56 ± 8.83	−43%	+2%	+2%
		Female	25.23 ± 8.22	25.04 ± 7.74	−19%	−5%	−6%
Control	3rd	Male	28.31 ± 10.87	27.49 ± 10.92	−82%	−2%	No change
		Female	25.61 ± 8.35	25.99 ± 8.12	+38%	No change	No change
	4th	Male	23.05 ± 10.09	23.54 ± 10.30	+49%	+6%	−17%
		Female	25.29 ± 8.83	25.52 ± 8.59	+23%	+4%	−11%

“Δ BF%” denotes the change in body fat percentage from baseline to follow-up. “+” indicates an increase and “−” indicates a decrease in percentages from pre- to post-assessment measurements.

## Data Availability

The original contributions presented in this study are included in the article. Further inquiries can be directed to the corresponding author.
